# A Patient-Centered Approach to Communication during Endoscopic Procedures: The Importance of Providing Information to Patients

**DOI:** 10.3390/ejihpe14060111

**Published:** 2024-06-09

**Authors:** Osnat Bashkin, Rita Boltean, Revaya Ben-Lulu, Mor Aharon, Ruhama Elhayany, Avraham Yitzhak, Revital Guterman, Naim Abu-Freha

**Affiliations:** 1 Department of Public Health, Ashkelon Academic College, Ben-Tzvi 12, Ashkelon 78211, Israel; 2 Assuta Medical Center, Beer-Sheva 8489507, Israel; ruhama@assuta.co.il (R.E.); aviyi@assuta.co.il (A.Y.); revitalo@assuta.co.il (R.G.);

**Keywords:** endoscopy, patient experience, pain, satisfaction, information, communication

## Abstract

The study aimed to explore patients’ experiences and perceptions throughout the various stages of endoscopic procedures and examine the association between patient-centered communication and the patient’s experience. A total of 191 patients responded to pre- and post-procedure surveys that inquired about fear and pain, patients’ satisfaction regarding the information provided to them, perceptions and experience. Pain was associated with post-procedure fear (r = 0.63, *p* < 0.01) and negatively associated with reported patient experience at the end of the visit (r = −0.17, *p* < 0.01). Significant positive associations were found between patient experience and satisfaction from the information provided before (r = 0.47, *p* < 0.01) and the information provided after the procedure (r = 0.51, *p* < 0.001). A predictive model found that perceptions toward the physicians, satisfaction from information provided before discharge, and feelings of trust are predictors of the patient experience (F = 44.9, R^2^ = 0.61, *p* < 0.001). Patients’ satisfaction with information provided before and after the procedure can positively affect the patients’ experience, leading to a decrease in fear and anxiety and increasing compliance with medical recommendations. Strategies for PCC with endoscopic patients should be developed and designed in a participatory manner, taking into account the various aspects associated with the patient experience.

## 1. Introduction

Endoscopic procedures performed in gastroenterology institutes are invasive procedures carried out in a one-day hospital stay and usually require preliminary preparation, anesthesia/sedation, and detailed instructions related to possible adverse events after discharge. Endoscopic examinations, such as gastroscopy and colonoscopy, are commonly performed by gastroenterologists for various indications. Both are relatively safe procedures with a low rate of complications [1].

Various quality indicators for performing an endoscopic procedure include the quality of colon preparation, the rate of reaching the end of the colon, the exit time during colonoscopy, and the polyp detection rate [2]. In recent years there has been increasing interest in patient-reported outcomes and the patient experience in the gastroenterology diagnostic procedure and treatment [3,4]. Nevertheless, many studies focused on procedure performance measures rather than analyzing significant patient-centered factors influencing patient experience [5].

According to the European Society of Gastrointestinal Endoscopy, patient experience is considered an important quality indicator of performing an invasive procedure [6], as it may significantly affect compliance rates of high-risk groups. Past studies mentioned various factors shaping the patient experience. Among these factors are the colonoscopy unit environment, perceptions of the medical teams’ technical skills, and long waiting times [7,8]. In addition, previous studies showed that the endoscopic procedure may cause significant fear and anxiety among patients which affects the patients’ experience [9,10]. Fear is a commonly reported barrier to undergoing a colonoscopy, followed by the unpleasant required preparation, lack of knowledge, and pain during the procedure [11,12]. 

Patients who need an endoscopic procedure often report worries and concerns before the procedure, mainly due to the lack or inaccuracy of knowledge about the endoscopic procedure. Lack of necessary information provided to the patient damages the patient–medical team communication, decreases patient cooperation and tolerance before and during the procedure, and can negatively affect the endoscopist’s performance [13,14]. Correct and adequate communication and information sharing by a professional team before and after the procedure is necessary to reduce worries and improve patient experience [8]. 

Patient–physician communication plays an important role in achieving positive outcomes, including patient satisfaction, patient–provider trust, adherence to physicians’ recommendations, and self-management of health conditions [15,16]. According to Epstein et al. [16], patients’ values and preferences should be at the forefront of the medical encounter through patient-centered communication (PCC). 

The PCC process involves attentively listening to patients’ concerns, demonstrating empathy, explaining and clarifying medical terms, as well as involving patients in the decision-making process, which in turn enhances trust, satisfaction and positive perceptions of the quality of healthcare [17,18,19]. 

There is a strong link between PCC and patient experience [20]. Patients often experience stress, anxiety, helplessness, and uncertainty related to their health status. During situations of uncertainty, through all stages of interaction with medical staff, patients seek information and interactive, reciprocal communication that may decrease discomfort [21]. The physician’s early provision of information to the patient, in addition to nonverbal expressiveness of empathy, reduces the patient’s uncertainty and anxiety during the medical encounter [20,22]. In addition, past studies found that task-oriented behaviors such as active listening and providing detailed information during the physician–patient interaction were strongly associated with patient satisfaction [23,24]. Patients often have limited abilities to evaluate medical processes; therefore, they assess their experience and satisfaction with the care they receive based on the quality of the communication with healthcare professionals [25,26]. Research also demonstrated the strong influence of PCC on trust in the patient–physician relationship [27], which in turn increases patient satisfaction [28]. Therefore, effective, patient-centered communication acts enhance positive patient experience [26]. 

Despite the importance of effective communication, especially when it comes to invasive medical procedures, few studies describe communication processes between medical teams and patients during endoscopic procedures. The experience of gastroscopy and colonoscopy procedures can be stressful for patients. While the medical benefits of these procedures are well-established, the potential for pain and fear can deter individuals from seeking out this care. Therefore, it is essential to understand the patient’s experience regarding these procedures and the various associated factors, mainly communication processes. 

This paper describes research aimed at exploring the patient’s experience and perceptions throughout the stages of endoscopic procedures. We hypothesized that patients’ satisfaction with information provided before and after the procedure will be positively associated with perceptions toward the endoscopic team and patients’ experience.

## 2. Materials and Methods

The study was approved by the Assuta Medical Center Ethics Committee (Approval # ASMC-0086-21). The study involved a survey of patients scheduled to undergo an endoscopic procedure at the endoscopic unit. 

### 2.1. Procedure and Population Sample

This cross-sectional study was carried out at the Beer-Sheva Assuta Endoscopic Unit in the southern region of Israel between March 2022 and December 2022. 

One hundred ninety-one patients above 18 years of age with a scheduled appointment to perform a standard endoscopic procedure (gastroscopy and colonoscopy) participated in the study. The exclusion criteria included inability to communicate or age less than 18. Participation was based on volunteering, and participants were informed about anonymity, data protection, and privacy and asked to sign a consent form. In the waiting room and before entering the procedure, patients received a hard copy of part A of the survey (pre-procedure survey), while at the end of the visit and before discharge, they received a hard copy of part B of the survey (post-procedure survey). (See Supplementary Material S1). The procedures lasted an hour on average and all the participants were given sedation during the procedures. 

### 2.2. Measures

The Part A pre-procedure survey comprised the following:

1. Demographics and clinical details: age, sex, marital status, employment status, referral doctor, examination type, whether it is the first time the patient is going through an endoscopic procedure, the indication for performing the examination, satisfaction from waiting time to receiving an appointment to the procedure.
2. Details regarding the required preparation for the procedure: respondents were asked to rate difficulty levels in preparing for the procedure as required by the guidelines on a 10-point scale (1—“not at all” to 10—“very hard”) and explain why it was difficult.
3. Feelings of fear from the procedure: respondents were asked to rate their fear levels on a 10-point scale (1—“not at all” to 10—“very afraid”) and explain the main reason for their concerns.
4. Details regarding the patient’s satisfaction with the information they received before the procedure: this part included eight statements describing information provided to the patient on the reason to perform the procedure, the preparation process, alternatives to the procedure, potential complications, the effect of the procedure on the patients’ health condition, and perceptions toward the quality of the information provided and the feeling of being involved in decision making. Respondents were asked to rate levels of agreement with each statement on a 5-point scale (1—“strongly disagree” to 5—“strongly agree”). (Reliability was measured using Cronbach’s alpha, α = 0.857.)

The Part B post-procedure survey comprised the following:

1. Details regarding waiting time for the procedure to start: respondents were asked to note how much time (in minutes) they waited in the waiting room and to rate the waiting time on a 5-point scale (1—“bad” to 5—“excellent”).
2. Details regarding feelings of pain and fear after undergoing the procedure: respondents were asked to rate levels of fear they felt after undergoing the procedure on a 10-point scale (1—“not at all” to 10—“very afraid”), and to rate the levels of pain they felt during the procedure and after the end of the procedure on a 10-point scale (1—“not at all” to 10—“very painful”). In addition, respondents were asked to mention what could have reduced their feelings of fear and concerns before undergoing the procedure.
3. Details regarding the patient’s experience, feelings of trust, perceptions toward the medical team, the patients’ satisfaction with the information they received in the procedure room moments before starting the procedure, satisfaction with the information they received after the end of the procedure (before discharge), and their general experience: respondents were asked to rate their levels of agreement with 11 statements on a 5-point scale (1—“strongly disagree” to 5—“strongly agree”), and to rate their general experience and perceptions with another five statements on a 5-point scale (1—“very bad” to 5—“excellent”). (Reliability was measured using Cronbach’s alpha, α = 0.852).

### 2.3. Statistical Analysis

Patient characteristics were presented as mean ± SD for continuous variables and percentages for categorical variables. Categorical variables were compared using the chi-square test. Data analysis was carried out using IBM SPSS Statistics 25.0 software. Reliability was measured using Cronbach’s alpha. An analysis of descriptive statistics was conducted to explore the demographics and characteristics of the respondents. Correlations were applied between patient clinical characteristics and pre-procedure fear, as well as the perceived difficulty of procedure preparation. *t*-tests for independent samples and one-way ANOVA were applied to examine differences in perceptions among sociodemographic groups; the post hoc evaluation was calculated using Tukey’s method. Pearson correlations were calculated to examine the association between variables. Finally, a model of linear regression was constructed to predict patient experience. *p* < 0.05 was accepted as significant.

## 3. Results

Of the 191 patients who responded to both parts of the survey, 99 were women (51.8%), and the mean age of respondents was 49.3 ± 14.6 years. Participants reported an average of 2.3 ± 2.4 weeks of waiting for a procedure appointment. Table 1 presents the characteristics of the sample of survey respondents.

Table 2 presents the differences in the study variables related to feelings of fear, pain, and difficulty in preparing for the procedure, as reported by patients among sociodemographic and clinical groups. 

As seen in Table 2, the analysis revealed that women, respondents who have been through the procedure before, and respondents undergoing both colonoscopy and gastroscopy at the same appointment expressed significantly higher levels of fear before the procedure. 

Further analysis revealed that respondents who reported a positive fecal occult blood test (FOBT) expressed significantly higher levels of fear before the procedure [Mean 6.2 ± 3.2, *t*-test = −2.5, *p* = 0.007] and significantly higher levels of fear after the procedure was ended [Mean 3.8 ± 2.8, *t*-test = −1.9, *p* = 0.029], compared to other reported reasons for undergoing the procedure. In addition, respondents who reported constipation expressed significantly higher levels of difficulty in preparing for the procedure [Mean 7.2 ± 3.6, *t*-test = −1.6, *p* = 0.048].

As for the reasons for feeling fear before the procedure, respondents mentioned fear of the procedure technique (36%), fear of anesthetics (18%), and fear of the procedure results (42%).

Table 3 presents the differences in the study variables related to patients’ satisfaction with the information provided, patients’ experience, and perceptions toward the medical team among sociodemographic and clinical groups.

As seen in Table 3, the analysis revealed that respondents’ experience was rated higher among those going through colonoscopy and those going through both colonoscopy and gastroscopy simultaneously, compared to respondents going through gastroscopy. In addition, respondents’ experience was rated higher among those going through the procedure for the first time. 

Table 4 shows the correlations between the survey variables. The analysis results revealed positive associations between difficulty levels in preparing for the procedure and feelings of fear before (r = 0.38) and after the procedure (r = 0.17). In addition, positive associations were also found between feelings of fear before the procedure and feelings of fear after the procedure (r = 0.49). Negative associations were found between perceptions of pain and patient experience (r = −0.17), perceptions toward physicians (r = −0.22) and nurses (r = −0.16), and feelings of trust (r = −0.15). Significant positive associations were found between satisfaction with the information provided before and the information provided after the procedure and patient experience, perceptions toward physicians and nurses, and feelings of trust.

Paired sample *t*-test revealed significant differences in the reported pain during and after the procedure and in the reported fear before and after the procedure. The analysis showed that perceptions of pain were higher after the procedure was ended, while fear was lower after the procedure was completed. In addition, respondents were more satisfied with the information provided after the procedure ended than before. Table 5 presents the analysis results.

Respondents mentioned several factors that they thought could reduce their fear: watching a guiding video before the procedure (8.4%), receiving sedatives (9.4%), receiving more explanation about the procedure (17%), allowing family members to be present in the procedure room (23%), and a personal conversation with the medical team before starting the procedure (28%). 

Table 6 shows a multiple regression model for study variables as predictors of patient experience. The model in Table 6 included only variables significantly contributing to the prediction. The analysis of the assumed regression model shows that perceptions toward the physicians, satisfaction with information before discharge, and feelings of trust are predictors of the patient experience. The variance explained by the final model was 61.0% of patient experience (*p* < 0.001).

## 4. Discussion

The current study aimed to thoroughly understand the patient’s experience before and after an endoscopic procedure in its various aspects and the factors that influence this experience, mainly patient–medical team communication elements during the medical encounter. Findings showed that the fear level among patients was high before the procedure, and decreased after the procedure was ended. However, pain assessments were higher after the procedure was ended among all patients who participated in the study. The pain was negatively associated with the patient experience reported at the end of the visit. These findings align with a recent study that examined 123 patients referred for colonoscopy and found half of the patients were above the cut-off for anxiety before the procedure. Moreover, the researchers found that notwithstanding sedation, behavioral manifestations of pain during colonoscopy indicated probable or moderate pain for about one-third of the patients [12]. 

Further to these findings, patients’ satisfaction with information provided before and after the procedure showed significant positive associations with patients’ experience, perceptions toward the endoscopic team, and feelings of trust. The positive effect of providing information on the patient’s perception of endoscopy, compliance with the procedure, and anxiety level associated with the procedure was previously presented in a randomized controlled trial among 300 patients [29]. Researchers found that 80.3% wished to know the possible risks associated with the procedure, and 93.3% wanted an explanation about the procedure (77.5% preferred a verbal explanation of the procedure). This research also demonstrated that patients who received detailed verbal information about the procedure experienced less pain, felt better during the procedure, were more satisfied, evaluated the procedure as less difficult, and showed better compliance with the procedure and a lower mean anxiety score compared to patients who received written information or no information at all. Similarly, in our study, 28% of patients mentioned that personal conversation with the endoscopic team before starting the procedure could reduce fear of the procedure, and 17% of the patients noted that receiving more information about the procedure could also have a positive effect on their fear and worries before the procedure. 

Evidence from past studies emphasizes the significant elements of PCC to meet the needs of patients to receive detailed information about common and uncommon risks associated with the procedure, the technique of the procedure, the expected feeling during and after it, benefits, and alternatives to the procedure [29,30,31]. Pre-procedure office visits that provide details about the procedure and allow patients to express concerns and ask questions have been associated with higher patient satisfaction [32]. 

Through PCC processes, providing information to patients can also increase the feeling of trust, which is necessary for patients’ compliance with invasive examinations. Our prediction model showed that feelings of confidence in the endoscopic team, satisfaction with the information provided after the procedure and before discharge, and perceptions towards the physicians (which mainly focused on their technical, interpersonal, and professional skills as seen through the eyes of the patients) are significant predictors of the patient experience. As confirmed recently by a previous study, a trusting relationship between a patient and a provider, built on PCC, is a significant factor in the patient evaluation of healthcare quality [19]. Therefore, our findings shed light on the elements of the patient’s experience and their needs. An in-depth examination of the patient’s experience can help improve the medical service at the level of the endoscopy team–patient encounter in the clinic, during the preparation and the performance of the endoscopic procedure, and at the post-procedure follow-up level. 

Limitations must be considered in the interpretation of the findings. First, this was a voluntary study based on self-reporting, and the data were subject to recall and selection bias. Another limitation is that the feedback was not analyzed by the severity of the condition, which may be another factor influencing the patient experience. In addition, all our data were collected from endoscopic procedures performed at a single center in the Israeli context; therefore, the findings may not be generalized to other countries with their distinct health delivery systems, comprising unique cultural and organizational characteristics, and within different clinical settings. However, we do believe the overall methodological research design may apply and be generalizable to other endoscopic centers. 

## 5. Conclusions

**Figure 1:**
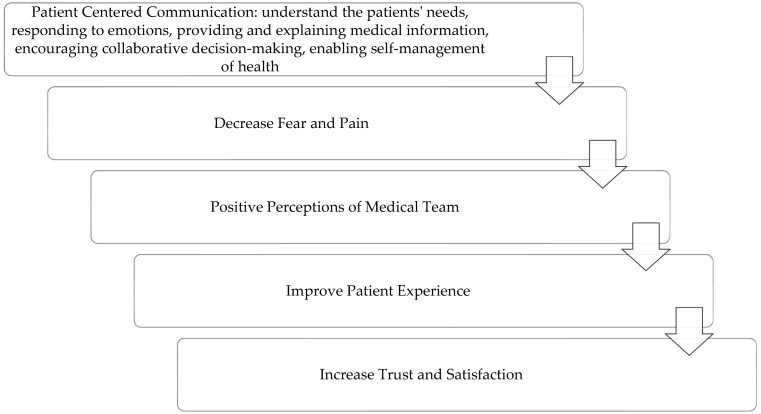
Patient-Centered Communication process and effect on patients’ perceptions and experiences in endoscopic procedures.

Endoscopic procedures are interventional procedures that may cause fear and stress for patients. Patients’ satisfaction with information provided before and after the procedure can positively affect the patients’ experience, which in turn leads to a decrease in fear and anxiety associated with the procedure and increase future compliance with medical recommendations related to endoscopic procedures. This study yielded important findings that need to be addressed to improve PCC processes and enhance the patients’ experience. Strategies for PCC with endoscopic patients should be developed and designed in a participatory manner, taking into account the various aspects associated with the patient experience. Best practices based on PCC skills for endoscopic procedures need to be formulated to include gathering information about the patients’ needs, responding to emotions, providing and explaining medical information, encouraging collaborative decision-making, and enabling self-management of health and care. It is essential to develop a structured framework for providing adequate and detailed patient-focused information to patients in all stages of the endoscopic process: pre-procedure, during the procedure, and post-procedure. Giving detailed information can have a positive effect on the patient’s experience, perception, compliance, and anxiety level associated with the procedure. Figure 1 summarizes the key findings and conclusions of the analysis. 

## Figures and Tables

**Table 1:** Characteristics of the study sample (N = 191).

Demographic and Clinical Variables		N (%)
Gender	Male	92 (48.2%)
	Female	99 (51.8%)
In a relationship	Yes	136 (71.2%)
	No	55 (28.8%)
Work status	Working	147 (76.9%)
	Not working	44 (23.1%)
Referral doctor	Family doctor	113 (59.2%)
	Gastro	78 (40.8%)
Examination type	Colonoscopy	73 (38.2%)
	Gastroscopy	62 (32.5%)
	Both	56 (29.3%)
Examination before	Yes	72 (37.7%)
	No	119 (62.4%)
Procedure’s indication	Abdominal pain	75 (39.3%)
	Heartburn	46 (24.1%)
	Positive FOBT ^1^	27 (14.1%)
	Changes in bowel habits	14 (7.3%)
	Constipation	13 (6.8%)
	Diarrhea	12 (6.3%)
	IBD ^2^ Follow-up	14 (7.3%)
	Cancer Follow-up	9 (4.7%)

^1^ IBD = Inflammatory bowel disease, ^2^ FOBT = Fecal occult blood test.

**Table 2:** Analysis of variance for comparisons between demographic and clinical groups regarding feelings of fear and pain (N = 191).

Demographic and Clinical Variables (N)		Level of Difficulty in Preparing for the Procedure M (SD)	Feelings of Fear before the Procedure M (SD)	Feelings of Pain during Procedure M (SD)	Feelings of Pain after the Procedure M (SD)	Feelings of Fear after Procedure M (SD)
Gender	Male (N = 92)	5.0 (2.9)	4.2 (2.7)	1.5 (1.3)	1.5 (1.1)	2.7 (2.3)
	Female (N = 99)	5.8 (3.1)	5.4 (3.1)	1.2 (1.0)	1.8 (1.5)	2.9 (2.6)
		−1.5 *^a^*	−2.6 ^a^**	1.2 *^a^*	−1.6 *^a^*	−0.5 *^a^*
In a relationship	Yes (N = 136)	5.3 (2.9)	4.9 (2.9)	1.4 (1.2)	1.6 (1.3)	2.9 (2.5)
	No (N = 55)	5.9 (3.2)	4.5 (3.1)	1.3 (1.1)	1.9 (1.5)	2.6 (2.3)
		1.1 *^a^*	−1.0 *^a^*	−2.4 *^a^*	1.2 *^a^*	−0.7 *^a^*
Work status	Working (N = 147)	5.3 (2.9)	4.7 (2.9)	1.3 (1.2)	1.7 (1.4)	2.7 (2.4)
	Not working (N = 44)	5.9 (3.0)	5.2 (3.0)	1.5 (1.1)	1.5 (1.0)	3.2 (2.6)
		1.1 *^a^*	1.0 *^a^*	0.8 *^a^*	−0.9 *^a^*	1.2 *^a^*
Referral doctor	Family doctor (N = 113)	5.2 (2.8)	4.8 (2.9)	1.4 (1.2)	1.6 (1.4)	3.0 (2.6)
	Gastro (N = 78)	5.6 (3.3)	4.8 (3.0)	1.3 (1.1)	1.6 (1.2)	2.6 (2.3)
		−0.7 *^a^*	−0.2 *^a^*	0.6 *^a^*	0.0 *^a^*	1.1 *^a^*
Examination type	Colonoscopy (N = 72)	5.2 (3.0)	5.1 (3.1)	1.2 (0.7)	1.4 (1.1)	2.9 (2.7)
	Gastroscopy (N = 62)	3.2 (2.4)	3.9 (2.7)	1.8 (1.6)	2.1 (1.5)	2.6 (2.1)
	Both (N = 56)	6.9 (2.5)	5.9 (2.7)	1.2 (0.9)	1.4 (1.0)	3.1 (2.6)
		11. 4 ^b^***	3. 15 ^b^*	3. 0 ^b^*	3. 6 ^b^*	0. 4 ^b^
Examination	Yes (N = 72)	5.1 (3.1)	5.6 (3.0)	1.2 (0.7)	1.4 (0.9)	3.2 (2.6)
before	No (N = 119)	5.7 (2.9)	4.3 (2.7)	1.5 (1.4)	1.8 (1.5)	2.7 (2.4)
		1.0 *^a^*	−3.0 ^a^**	1.5 *^a^*	2.2 ^a^*	−1.3 *^a^*

* *p* < 0.05, ** *p* < 0.01, *** *p* < 0.001. *^a^* *t*-test. ^b^ One-way ANOVA test. The scale ranged from 1 to 10.

**Table 3:** Analysis of variance for comparisons between demographic and clinical groups regarding patients’ satisfaction, experience, and perceptions toward the medical team (N = 191).

Demographic and Clinical Variables (N)		Satisfaction with Information before the Procedure M (SD)	Satisfaction with Information after the Procedure M (SD)	Patient Experience M (SD)	Perceptions toward Physicians M (SD)	Perceptions toward Nurses M (SD)	Feelings of Trust M (SD)
Sex	Male (N = 92)	4.2 (0.6)	4.6 (0.5)	4.5 (0.5)	4.8 (0.2)	4.8 (0.3)	4.7 (0.3)
	Female (N = 99)	4.1 (0.8)	4.7 (0.4)	4.6 (0.4)	4.8 (0.3)	4.8 (0.3)	4.8 (0.3)
		0.5 *^a^*	−1.1 *^a^*	−1.2 *^a^*	0.8 *^a^*	−0.5 *^a^*	−0.5 *^a^*
In a relationship	Yes (N = 136)	4.3 (0.7)	4.7 (0.4)	4.6 (0.4)	4.9 (0.4)	4.9 (0.3)	4.8 (0.3)
	No (N = 55)	3.8 (0.8)	4.6 (0.5)	4.5 (0.5)	4.8 (0.3)	4.8 (0.3)	4.7 (0.4)
		−2.9 ^a^**	−1.0 *^a^*	−1.3 *^a^*	−1.1 *^a^*	−0.6 *^a^*	−0.9 *^a^*
Work status	Working (N = 147)	4.1 (0.8)	4.6 (0.5)	4.6 (0.5)	4.9 (0.3)	4.9 (0.3)	4.8 (0.4)
	Not working (N = 44)	4.2 (0.7)	4.7 (0.4)	4.6 (0.4)	4.8 (0.4)	4.8 (0.4)	4.7 (0.4)
		−0.2 *^a^*	0.3 *^a^*	0.1 *^a^*	−0.8 *^a^*	−0.8 *^a^*	−0.6 *^a^*
Referral doctor	Family doctor (N = 113)	4.0 (0.7)	4.6 (0.4)	4.6 (0.4)	4.8 (0.4)	4.9 (0.3)	4.8 (0.3)
	Gastro (N = 78)	4.2 (0.8)	4.7 (0.5)	4.6 (0.5)	4.9 (0.3)	4.8 (0.4)	4.8 (0.4)
		−0.8 *^a^*	−0.6 *^a^*	−0.5 *^a^*	−0.3 *^a^*	0.6 *^a^*	0.1 *^a^*
Examination type	Colonoscopy (N = 72)	4.0 (0.7)	4.6 (0.5)	4.7 (0.4)	4.8 (0.3)	4.8 (0.4)	4.8 (0.3)
	Gastroscopy (N = 62)	4.3 (0.6)	4.6 (0.4)	4.5 (0.6)	4.9 (0.2)	4.9 (0.3)	4.8 (0.4)
	Both (N = 56)	4.0 (0.8)	4.7 (0.5)	4.7 (0.4)	4.8 (0.4)	4.8 (0.2)	4.7 (0.4)
		2.2 ^b^	0.3 ^b^	2.9 ^b^*	2.5 ^b^	2.1 ^b^	0.3 ^b^
Examination	Yes (N = 72)	4.2 (0.7)	4.7 (0.4)	4.7 (0.4)	4.9 (0.2)	4.8 (0.3)	4.9 (0.2)
before	No (N = 119)	4.1 (0.8)	4.6 (0.5)	4.5 (0.6)	4.8 (0.3)	4.8 (0.3)	4.7 (0.4)
		−0.4 *^a^*	−1.3 *^a^*	−2.1 ^a^*	−2.2 ^a^*	−0.6 *^a^*	−3.1 ^a^***

* *p* < 0.05, ** *p* < 0.01, *** *p* < 0.001. *^a^* *t*-test. ^b^ One-way ANOVA test. The scale ranged from 1 to 5.

**Table 4:** Pearson correlations between the survey variables (N = 191).

	Feelings of Fear before Procedure	Feelings of Pain during Procedure	Feelings of Pain after Procedure	Feelings of Fear after Procedure	Satisfaction with Information before Procedure	Satisfaction with Information after Procedure	Patient Experience	Perceptions toward Physicians	Perceptions toward Nurses	Feelings of Trust
Level of difficulty in preparing for the procedure	0.38 **	0.03	0.18	0.17 *	−0.16	−0.01	−0.07	−0.18 *	.12	−0.10
Feelings of fear before procedure	---	−0.14	0.12	0.49 **	−0.02	0.06	0.08	0.00	−0.11	0.00
Feelings of pain during procedure	---	---	0.63 **	0.14	0.06	−0.04	−0.06	−0.30 **	−0.09	−0.05
Feelings of pain after procedure	---	---	---	0.16 *	0.00	−0.12	−0.17 *	−0.22 **	−0.16 *	−0.15 *
Feelings of fear after procedure	---	---	---	---	−0.02	−0.18 *	0.01	−0.07	−0.06	−0.10
Satisfaction with information before procedure	---	---	---	---	---	0.46 ***	0.47 ***	0.46 ***	0.26 **	0.51 ***
Satisfaction with information after procedure	---	---	---	---	---	---	0.51 **	0.55 **	0.35 ***	0.56 ***
Patient experience	---	---	---	---	---	---	---	0.46 ***	0.51 ***	0.57 ***
Perceptions toward physicians	---	---	---	---	---	---	---	---	0.38 ***	0.47 ***
Perceptions toward nurses	---	---	---	---	---	---	---	---	---	0.43 ***

* *p* < 0.05, ** *p* < 0.01, *** *p* < 0.001.

**Table 5:** Analysis of paired sample *t*-test for comparing feelings of fear and pain before, during, and after the procedure (N = 191).

		Mean (SD)	T	*p*
Pain	Pain during	1.4 (1.2)	−3.4	0.001
	Pain after	1.7 (1.4)		
Fear	Fear before	4.8 (3.0)	9.4	0.001
	Fear after	2.8 (2.5)		
Information	Information before	4.2 (0.8)	−4.9	0.001
	Information after	4.7 (0.5)		

**Table 6:** Multiple regression model for study variables as predictors of patient experience.

Dimension/Variable	B	Beta	*p*
Perceptions toward the physicians	0.40	0.30	0.001
Satisfaction with information before discharge	0.25	0.32	0.004
Trust	0.41	0.28	0.001

## Data Availability

The data that support the findings of this study are available from the corresponding author.

## Supplementary Materials

The following supporting information can be downloaded at https://www.mdpi.com/article/10.3390/ejihpe14060111/s1, S1: Study surveys.
