# Supplementary material for: A Patient-Centered Approach to Communication during Endoscopic Procedures: The Importance of Providing Information to Patients
Source: Eur J Investig Health Psychol Educ. 2024 Jun 9;14(6):1688–99. doi: 10.3390/ejihpe14060111 (PMC11202659; doi:10.3390/ejihpe14060111)
Supplement: Supplementary file 1 [file ejihpe-14-00111-s001.zip › ejihpe-3032285-supplementary.pdf]

Supplementary Material - Surveys

Part A: Pre-procedure survey

1. Gender: *1. Male 2. Female*
2. Age: ___
3. Marital status: *1. In a relationship 2. Not in a relationship*
4. Employment status: *1. Working 2. Not working*
5. Please Indicate your referral doctor: *1. Family doctor 2. Gastroenterologist*
6. Examination type: *1. Colonoscopy 2. Gastroscopy 3. Both*
7. Have you been through an endoscopic examination before? *1. Yes 2. No*
8. Please indicate the reasons you were referred for the procedure:
   a) *Abdominal pain*
   b) *Heartburn*
   c) *Positive FOBT*
   d) *Changes in bowel habits*
   e) *Constipation*
   f) *Diarrhea*
   g) *Monitoring of IBD*
   h) *Monitoring of cancer*
9. Please Indicate your satisfaction level regarding the waiting time to receive an appointment to the procedure: *Bad, Reasonable, Good, Very good, Excellent.*
10. Please rate the level of difficulty you felt in preparing for the procedure as required:

1- not at all……………………………………………………………......10 - very hard

11. Please indicate the reason it was difficult: ________________________________
12. Please rate the level of fear you feel now:

1- not at all……………………………………………………………......10 - very afraid

13. Please indicate the reason you are afraid: ________________________________

Circle the number indicating the extent to which you agree with the following statements:

	**Strongly disagree**				**Strongly agree**
1. At the clinic the medical staff gave me clear information about the reason for my referral to perform the examination	1	2	3	4	5

2. At the clinic, I was given clear information about how to prepare for the procedure	1	2	3	4	5
3. At the clinic, I was given clear information about the optional alternatives for the procedure	1	2	3	4	5
4. At the clinic, I was given clear information about the possible complications of the procedure	1	2	3	4	5
5. At the clinic, I was given clear information about the possible effect of the procedure on my health condition	1	2	3	4	5
6. The information I received before the procedure was explained to me in a clear, comprehensive, and satisfying way.	1	2	3	4	5
7. I feel that I was involved in making the decision about performing the procedure and the necessary preparations for it.	1	2	3	4	5
8. My questions were answered patiently by the staff at the clinic	1	2	3	4	5

Part B: Post-procedure survey (after discharge from the unit)

1. Please Indicate how much time (in minutes) you waited in the waiting room before the procedure: __________________
2. Please Indicate your satisfaction level regarding the waiting time to enter to the procedure room: Bad, Reasonable, Good, Very good, Excellent.
3. Please rate the level of pain you felt during the procedure:

1- not at all……………………………………………………………......10 - very painful

14. Please rate the level of pain you feel now at the end of the procedure:

1- not at all……………………………………………………………......10 - very painful

4. Please rate the level of fear you feel now at the end of the procedure:

1- not at all……………………………………………………………......10 - very afraid

5. Please indicate what could have reduced your feeling of fear and concerns before the procedure:
   a) *Watching a guiding video before the procedure*
   b) *Receiving sedatives*
   c) *Receiving more explanation about the procedure*
   d) *Allowing family member to be present in the procedure room*
   e) *A personal conversation with the medical team before starting the procedure.*

Circle the number indicating the extent to which you agree with the following statements:

	Strongly disagree				Strongly agree
1. The environment of the waiting room where I waited for the procedure was comfortable and pleasant	1	2	3	4	5
2. The meeting with the doctor in the minutes before the procedure was pleasant and I felt comfortable asking questions	1	2	3	4	5
3. Before sedation, I received an additional explanation about the examination process	1	2	3	4	5
4. My concerns were taken seriously by the staff, and I felt that they listened to me	1	2	3	4	5
5. I felt confident in the team who was treating me during the examination	1	2	3	4	5
6. The treatment plan and follow-up were clearly outlined before I was released	1	2	3	4	5
7. I was informed of the potential side effects and possible complications of the procedure before I was released	1	2	3	4	5
8. Before I was released, my questions were answered patiently by the staff at the clinic	1	2	3	4	5
9. In my opinion, the physician who treated me was highly skilled and professional	1	2	3	4	5
10. In my opinion, the nurses who treated me were highly skilled and professional	1	2	3	4	5
11. I have had a positive experience in the endoscopic unit during my stay	1	2	3	4	5
**According to my impression:**	**Very bad**				**Excellent**
1. During my stay at the unit and during the procedure, the physician's communication style was:	1	2	3	4	5
2. During the procedure, the physician's technical skills were:	1	2	3	4	5
3. How were the results of your examination explained to you before you were released?	1	2	3	4	5
4. During my stay at the unit and during the procedure, the nursing staff communication style was:	1	2	3	4	5
5. Your general experience in the endoscopic unit is:	1	2	3	4	5

Thank you for your cooperation!
